# Supplementary material for: Identification of Potential Biomarkers and Biological Pathways for Poor Clinical Outcome in Mucinous Colorectal Adenocarcinoma
Source: Cancers (Basel). 2021 Jun 30;13(13):3280. doi: 10.3390/cancers13133280 (PMC8268122; doi:10.3390/cancers13133280)
Supplement: Supplementary file 1 [file cancers-13-03280-s001.zip › cancers-1277913-supplementary.pdf]

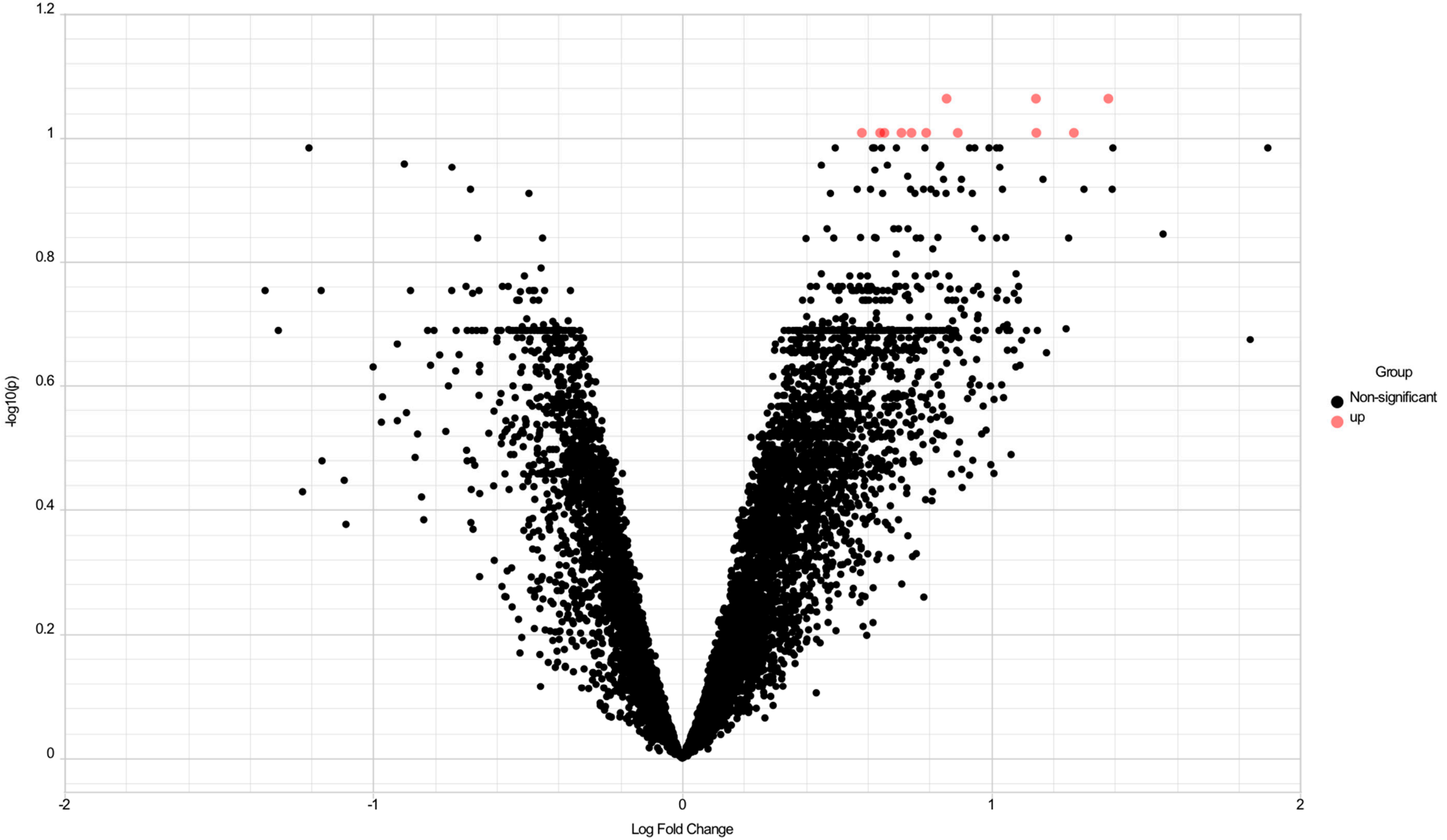

Figure S1. Volcano plot of differentially expressed genes between high risk- and low risk- group in mucinous colorectal cancer.

Supplementary Table S1. Baseline characteristics before/after propensity score matching.

	**Before matching**			**After matching**		
	**MAC**	**TAC**	**p-value**	**MAC**	**TAC**	**p-value**
Stage I						
Total	1347	7324		1342	1342	
Age at diagnosis, y, mean (SD)	71.0 (12.9)	66.8 (12.5)	<0.001	71.0 (12.9)	71.4 (12.1)	0.381
Sex, n (%)			<0.001			0.389
Male	576 (42.8)	3677 (50.2)		574 (42.8)	551 (41.1)	
Female	771 (57.2)	3647 (49.8)		768 (57.2)	791 (58.9)	
Tumor grade, n (%)			<0.001			0.259
Well-differentiated	236 (17.5)	1766 (24.1)		236 (17.6)	210 (15.6)	
Moderately-differentiated	957 (71.0)	5178 (70.7)		955 (71.2)	1001 (74.6)	
Poorly-differentiated	134 (9.9)	335 (4.6)		132 (9.8)	115 (8.6)	
Undifferentiated	20 (1.5)	45 (0.6)		19 (1.4)	16 (1.2)	
Race, n (%)			<0.001			0.969
Hispanic (All Races)	136 (10.1)	723 (9.9)		135 (10.1)	139 (10.4)	
Non-Hispanic American Indian/Alaska Native	4 (0.3)	30 (0.4)		4 (0.3)	3 (0.2)	
Non-Hispanic Asian or Pacific Islander	70 (5.2)	510 (7.0)		70 (5.2)	71 (5.3)	
Non-Hispanic Black	131 (9.7)	1065 (14.5)		131 (9.8)	122 (9.1)	
Non-Hispanic White	1006 (74.7)	4996 (68.2)		1002 (74.7)	1007 (75.0)	
Tumor size, mm, mean (SD)	37.3 (42.4)	23.7 (20.3)	<0.001	35.7 (21.8)	35.3 (24.0)	0.665

Stage II

Total	4390	2704		2618	2618	
Age at diagnosis, y, mean (SD)	69.8 (14.5)	68.6 (13.9)	<0.001	68.8 (14.7)	68.9 (13.8)	0.877
Sex, n (%)			<0.001			0.934
Male	2010 (45.8)	1326 (49.0)		1267 (48.4)	1263 (48.2)	
Female	2380 (54.2)	1378 (51.0)		1351 (51.6)	1355 (51.8)	
Tumor grade, n (%)			<0.001			0.151
Well-	522 (11.9)	341 (12.6)		335 (12.8)	326 (12.5)	
Moderate-	3144 (71.6)	2105 (77.8)		2040 (77.9)	2036 (77.8)	
Poor-	615 (14.0)	213 (7.9)		217 (8.3)	211 (8.1)	
Undifferentiated	109 (2.5)	45 (1.7)		26 (1.0)	45 (1.7)	
Race, n (%)			<0.001			0.917
Hispanic (All Races)	469 (10.7)	315 (11.6)		304 (11.6)	302 (11.5)	
Non-Hispanic American Indian/Alaska Native	12 (0.3)	10 (0.4)		8 (0.3)	7 (0.3)	
Non-Hispanic Asian or Pacific Islander	261 (5.9)	174 (6.4)		173 (6.6)	168 (6.4)	
Non-Hispanic Black	453 (10.3)	364 (13.5)		349 (13.3)	329 (12.6)	
Non-Hispanic White	3195 (72.8)	1841 (68.1)		1784 (68.1)	1812 (69.2)	
Tumor size, mm, mean (SD)	64.5 (42.4)	54.0 (47.6)	<0.001	54.3 (30.0)	53.3 (30.4)	0.211

Stage III						
Total	2286	1858		1492	1492	
Age at diagnosis, y, mean (SD)	67.05 (15.07)	64.98 (13.36)	<0.001	66.17 (14.87)	66.00 (13.43)	0.745
Sex, n (%)			0.4307			0.509
Male	1091 (47.7)	863 (46.4)		684 (45.8)	703 (47.1)	
Female	1195 (52.3)	995 (53.6)		808 (54.2)	789 (52.9)	

Tumor grade, n (%)			<0.001			0.883
Well-differentiated	166 (7.3)	159 (8.6)		113 (7.6)	119 (8.0)	
Moderately-differentiated	1428 (62.5)	1378 (74.2)		1073 (71.9)	1059 (71.0)	
Poorly-differentiated	538 (23.5)	247 (13.3)		230 (15.4)	242 (16.2)	
Undifferentiated	154 (6.7)	74 (4.0)		76 (5.1)	72 (4.8)	
Race, n (%)			<0.001			0.880
Hispanic (All Races)	309 (13.5)	206 (11.1)		166 (11.1)	180 (12.1)	
Non-Hispanic American Indian/Alaska Native	12 (0.5)	11 (0.6)		8 (0.5)	9 (0.6)	
Non-Hispanic Asian or Pacific Islander	173 (7.6)	145 (7.8)		125 (8.4)	115 (7.7)	
Non-Hispanic Black	284 (12.4)	320 (17.2)		209 (14.0)	215 (14.4)	
Non-Hispanic White	1508 (66.0)	1176 (63.3)		984 (66.0)	973 (65.2)	
Tumor size, mm, mean (SD)	62.08 (44.99)	44.45 (35.71)	<0.001	51.01 (27.00)	50.28 (27.97)	0.467

Stage IV						
Total	1073	604		595	595	
Age at diagnosis, y, mean (SD)	64.16 (14.41)	63.96 (13.87)	0.7857	63.87 (14.41)	64.08 (13.88)	0.800
Sex, n (%)			0.9698			0.816
Male	495 (46.1)	280 (46.4)		282 (47.4)	277 (46.6)	
Female	578 (53.9)	324 (53.6)		313 (52.6)	318 (53.4)	
Tumor grade, n (%)			<0.001			0.940
Well-differentiated	82 (7.6)	29 (4.8)		29 (4.9)	29 (4.9)	
Moderately-differentiated	644 (60.0)	422 (69.9)		418 (70.3)	413 (69.4)	
Poorly-differentiated	266 (24.8)	117 (19.4)		117 (19.7)	117 (19.7)	

	Undifferentiated	81 (7.5)	36 (6.0)		31 (5.2)	36 (6.1)	
Race, n (%)				<0.001			0.813
	Hispanic (All Races)	133 (12.4)	62 (10.3)		56 (9.4)	62 (10.4)	
	Non-Hispanic American Indian/Alaska Native	5 (0.5)	5 (0.8)		3 (0.5)	4 (0.7)	
	Non-Hispanic Asian or Pacific Islander	76 (7.1)	43 (7.1)		53 (8.9)	43 (7.2)	
	Non-Hispanic Black	157 (14.6)	133 (22.0)		129 (21.7)	126 (21.2)	
	Non-Hispanic White	702 (65.4)	361 (59.8)		354 (59.5)	360 (60.5)	
Tumor size, mm, mean (SD)		68.75 (58.62)	59.85 (45.63)	0.001	60.42 (25.22)	58.44 (25.58)	0.179

PSM, propensity score matching; MAC, mucinous adenocarcinoma; TAC, traditional adenocarcinoma; SD, standard deviation. Data represent frequency (percentage) or mean ± standard deviation, as appropriate.

Supplementary Table S2. Hazard ratios from Cox proportional hazards model between TAC and MAC.

Stage	Outcome	Type	HR	97.5% CIs	*P*-value
Stage I	Disease-specific survival	TAC	reference		
		MAC	1.052	(0.762 - 1.453)	0.757
	Overall survival	TAC	reference		
		MAC	1.038	(0.904 - 1.193)	0.597
Stage II	Disease-specific survival	TAC	reference		
		MAC	1.109	(0.960 - 1.281)	0.160
	Overall survival	TAC	reference		
		MAC	1.152	(1.051 - 1.263)	0.002
Stage III	Disease-specific survival	TAC	reference		
		MAC	1.428	(1.222 - 1.670)	<0.001
	Overall survival	TAC	reference		
		MAC	1.300	(1.137 - 1.486)	<0.001
Stage IV	Disease-specific survival	TAC	reference		
		MAC	1.222	(1.062 - 1.405)	0.005
	Overall survival	TAC	reference		
		MAC	1.182	(1.034 - 1.353)	0.015

TAC, traditional adenocarcinoma; MAC, mucinous adenocarcinoma; HR, hazard ratio; CIs, confidence intervals.

Supplementary Table S3. Significant genes for high risk prediction from differentially expressed gene in stage II mucinous colon cancer.

Symbols	EntrezID	logFC	AveExpr	P.Value	adj.P.Val	B	t
RPS18	6222	1.3792	8.7213	1.24E-05	0.08651	3.0186	5.0333
CENPL	91687	1.1448	6.2231	3.32E-05	0.08651	2.175	4.7148
RPL30	6156	1.146	8.6991	0.00010413	0.098192	1.1936	4.34
NME2	4831	1.2676	7.7072	0.00012542	0.098192	1.0339	4.2783
USP33	23032	0.85561	5.097	2.97E-05	0.08651	2.2717	4.7515
GAB2	9846	0.74188	6.4239	5.75E-05	0.098192	1.7036	4.5356
RPS3A	6189	0.58139	8.5389	6.55E-05	0.098192	1.5914	4.4927
RPS25	6230	0.70943	9.5035	0.00010222	0.098192	1.2095	4.3461
HNMT	3176	0.89175	4.9166	0.00011461	0.098192	1.1113	4.3082
CEP57	9702	0.64048	5.128	0.00012707	0.098192	1.0227	4.2739
ZC3H8	84524	0.65432	4.3263	0.00014427	0.098192	0.91366	4.2317
TRIT1	54802	0.78953	5.6654	0.0001507	0.098192	0.87626	4.2172

Supplementary Table S4. Tissue specific protein-protein interaction in network analysis.

Pathway	Total	Expected	Hits	P-value	FDR	Gene
Pathways in cancer	530	8.97	32	1.27E-10	1.01E-08	NOS2, ESR1, TP53, CUL1, NFKB2, FN1, CDK2, CUL2, EGFR, STK4, GRB2, CRK, CDKN1A, MET, PIK3CB, PLCG2, PLCG1, AKT1, RALA, BCR, CRKL, CTBP1, ESR2, MDM2, MYC, PIK3R1, RALB, TCEB1, TCEB2, TRAF6, VHL, RALBP1
Viral carcinogenesis	201	3.4	22	1.85E-12	5.89E-10	HIST1, H2BI, HIST1, H2BG, HIST1, H2BF, HIST1, H2BE, HIST1, H2BC, TP53, NFKB2, CDK2, SNW1, YWHAZ, GRB2, CDKN1A, HDAC6, YWHAE, YWHAQ, PIK3CB, YWHAH, YWHAB, LYN, MDM2, PIK3R1, YWHAG
PI3K-Akt signaling pathway	354	5.99	20	1.77E-06	2.66E-05	TP53, FN1, ITGA4, CDK2, EGFR, YWHAZ, GRB2, CDKN1A, YWHAE, YWHAQ, MET, PIK3CB, YWHAH, AKT1, YWHAB, MDM2, MYC, PIK3R1, SGK1, YWHAG
Proteoglycans in cancer	201	3.4	19	8.87E-10	4.70E-08	ESR1, TP53, FN1, ACTB, EGFR, GRB2, CDKN1A, IQGAP1, MET, PIK3CB, PLCG2, PLCG1, AKT1, PTPN11, PTPN6, GAB1, MDM2, MYC, PIK3R1
Ras signaling pathway	232	3.93	19	9.85E-09	3.48E-07	GAB2, EGFR, STK4, GRB2, MET, PIK3CB, PLCG2, PLCG1, AKT1, PTPN11, LAT, RALA, GAB1, PIK3R1, RAB5A, RALB, ZAP70, RALBP1, EXOC2

Supplementary Table S5. Key genes selected based on topological parameters like BC and degree.

Gene	Degree	Betweenness	Expression
RPS3A	48	11147.49	0.58139
CEP57	37	6679.16	0.64048
RPS18	35	3941.01	1.3792
RPL30	34	4002.75	1.146
GAB2	29	5054.56	0.74188
NME2	28	4816.77	1.2676
RPS25	26	1947.26	0.70943
USP33	17	3096	0.85561
